# Characterization of lncRNA/circRNA-miRNA-mRNA network to reveal potential functional ceRNAs in the skeletal muscle of chicken

**DOI:** 10.3389/fphys.2022.969854

**Published:** 2022-09-29

**Authors:** Zegun Pan, Chaowu Yang, Ruipeng Zhao, Xiaosong Jiang, Chunli Yu, Zhixiong Li

**Affiliations:** ^1^ Key Laboratory of Qinghai-Tibetan Plateau Animal Genetic Resource Reservation and Utilization of Ministry of Education Southwest Minzu University, Chengdu, Sichuan, China; ^2^ Animal Breeding and Genetics Key Laboratory of Sichuan Province, Sichuan Animal Science Academy, Chengdu, Sichuan, China

**Keywords:** chicken, ceRNA, lncRNA/circRNA, miRNA, skeletal muscle

## Abstract

Skeletal muscle, comprising approximately 40% of body mass, is a highly complex and heterogeneous tissue serving a multitude of functions in the organism. Non-coding RNAs (ncRNAs) are known to participate in skeletal muscle development as critical regulators. However, the regulatory mechanisms of ncRNAs on chicken muscle traits are not well understood. In the present study, we collected the leg muscle from male embryos of Tibetan chicken at embryonic (E) 10 and E18 for RNA sequencing. A total of 6,583 differentially expressed mRNAs (DEMs) including 3,055 down-regulated and 3,528 up-regulated were identified in E18. We identified 695 differentially expressed lncRNAs (DELs) (187 down-regulated and 508 up-regulated) and 1,906 differentially expressed circRNAs (DECs) (1,224 down-regulated and 682 up-regulated) in E18. Among the 130 differentially expressed miRNAs (DEMIs), 59 were up-regulated and 71 were down-regulated in E18. Numerous DEMs and target genes for miRNAs/lncRNAs were significantly enriched in the muscle system process and cell cycle. We constructed a miRNA-gene-pathway network by considering target relationships between genes related to skeletal muscle development and miRNAs. A competing endogenous RNA (ceRNA) network was also constructed by integrating competing relationships between DEMs, DELs, and DECs. Several DELs and DECs were predicted to regulate the ADRA1B, ATP2A2, ATP2B1, CACNA1S, CACNB4, MYLK2, and ROCK2 genes. We discovered the crosstalk between the ncRNAs and their competing mRNAs, which provides insights into ceRNA function and mechanisms in the skeletal muscle development of chicken.

## Introduction

The meat products developed by skeletal muscle have been one of the most important animal products for human consumption in livestock production. Mammalian skeletal muscle occupies approximately 40% of total body weight, which is essential for vital functions such as breathing, movement, and thermogenesis (Bentzinger et al., 2012; Dumont et al., 2015). Several levels of intrinsic complexity during myogenesis arise from hierarchical interactions between transcriptional regulators and regulatory RNAs in addition to other extrinsic regulators. The differentiation and formation of skeletal muscle entail activation of muscle-specific transcription network governed by muscle-specific regulatory factors (MRFs) (Delfini et al., 2000; Buckingham and Rigby, 2014). The impairment of skeletal muscle myogenesis is related in various muscle dysfunctions, including sarcopenia and muscular dystrophy (Chang et al., 2016; Cruz-Jentoft and Sayer, 2019). There are many studies show that skeletal muscle development is also significantly affected by post-transcriptional regulation (Cesana et al., 2011; Yue et al., 2019). However, the underlying molecular mechanism remains poorly understood.

Non-coding RNAs (ncRNAs) which are RNA molecules have little protein-coding capacity act as regulators in multifarious biological processes instead of transcriptional noise (Huarte, 2009; Mchugh et al., 2015). Recently, accumulating evidence has revealed that the ncRNAs including microRNA (miRNA), long non-coding RNA (lncRNA), and circular RNA (circRNA) regulated mRNA expression by functioning as a competing endogenous RNA (ceRNA) at the post-transcriptional level (Salmena et al., 2011). As non-protein-coding transcripts, lncRNAs range from 0.2 to 100 kb and have abundant binding sites for miRNAs (Phelps et al., 2016). For example, A newly identified lncRNA MAR1 promoted skeletal muscle differentiation and regeneration by acting as a miR-487b sponge (Zhang et al., 2018). LncIRS1 regulated muscle atrophy through sponging miR-15 family to activate IGF1-PI3K/AKT pathway in chicken (Li et al., 2019b). CircRNAs are a new type of endogenous ncRNAs that form closed continuous loops without 3′- and 5′- ends (Greene et al., 2017; Zeng et al., 2017). In recent years, there were many studies on the effect of circRNAs on the regulation of skeletal muscle myogenesis. For example, CircARID1A regulated mouse skeletal muscle regeneration by functioning as a sponge of miR-6368 (Liu et al., 2021). CircRNAs such as circTMTC1 (Shen et al., 2019), circFAM188B (Yin et al., 2020), circHIPK3 (Chen et al., 2019), and circSVIL (Ouyang et al., 2018) showed vital roles in myogenesis by acting as ceRNA in chicken. Only a few lncRNAs and circRNAs have been well annotated up to now. Our study aimed to establish an accurate regulatory mechanism based on the ceRNA theory in the skeletal muscle development of chicken.

The transcriptome is the set of all RNA molecules, including mRNA, lncRNA, circRNA, and miRNA produced in one or a population of cells (Liu et al., 2014). Unlike the genome, the transcriptome reflects the gene expression under certain physiological conditions or developmental stages (Liu et al., 2018). RNA-seq is a highly sensitive method for whole transcriptome analysis. In this study, we investigated the expression level of miRNAs, lncRNAs, circRNAs, and mRNAs in the skeletal muscle of Tibetan chicken by RNA-seq. Subsequently, we constructed the lncRNA/circRNA-miRNA-mRNA regulatory networks to identify the crucial factors involved in the development of chicken skeletal muscle.

## Materials and methods

### Experimental animals and sample collection

Fertilized eggs were obtained from Tibetan chickens (TC) were incubated under humid conditions at 37.5°C until they reached appropriate stages. On the day of harvest, the chicken embryos were sacrificed and the gender was determined according to the fetus anatomical characteristics. The leg muscle from six male embryos (three randomly selected embryos from each period) at embryonic (E) 10 and E18 were obtained, divided into three parts. Two parts for RNA-seq and real time quantitative PCR detection (RT-qPCR) were immediately frozen in liquid nitrogen and stored at −80°C, whereas the other part was fixed in 4% paraformaldehyde and embedded in paraffin for histological observation. All treatment procedures were approved by the Institutional Animal Care and Use Committee of the Southwest Minzu University.

### Histological examination of the chicken skeletal muscle

The histological characteristics in the skeletal muscle were evaluated by haematoxylin and eosin (H&E) staining. Paraffin sections were mounted on slides for H&E staining. Histological characteristics of the chicken skeletal muscle were observed using a BA210 Digital microscope (Motic) and Images Advanced Software (Motic). Two independent samples (E10 sample and E18 sample) were used in this experiment, and each of the samples had five biology repeats.

### Total RNA isolation and illumina sequencing

Total RNA was isolated from each sample using TRIzol reagent (Invitrogen™, Carlsbad, CA, United States) according to the manufacturer’s instructions. Integrity, purity, and quality of the isolated RNA were measured by agarose gel electrophoresis, Nano-Drop ND-2000 spectrophotometer (NanoDrop Products, Wilmington, DE, United States), and Agilent 2,100 Bioanalyzer (Agilent Technologies, Massy, France). The RNA integrity number (RIN) value of samples larger than eight were used for further analysis.

Approximately 9 μg of RNA per sample was used in the protocol to deplete ribosomal RNA (rRNA) by the Ribo-zero™ rRNA Removal kit (Epicentre, Madison, WI, United States). The rRNA-depleted RNAs were used for preparing the libraries following the manufacturer’s recommendations in the NEBNext® Ultra™ Directional RNA Library Prep Kit for Illumina (NEB, United States). The library fragments were purified with AMPure XP system (Beckman Coulter, Beverly, United States), and library quality was assessed on the Agilent Bioanalyzer 2,100 system. Paired-end sequencing with 125-bp reads was performed on the Illumina HiSeq 2,500 platform. A total of 3 μg of RNA per sample was used for the small RNA library. The libraries were also sequenced on an Illumina HiSeq 2,500 platform and 50-bp single-end reads were generated.

### RNA-seq data analysis

Clean data (clean reads) were obtained by removing the poor-quality bases and adapter sequences from the raw data using FastQC (v0.19.5), and all the downstream analyses were based on the clean data with high quality. The clean reads were aligned to the chicken genome sequence assembly (Genome Reference Consortium Chicken Build 6a, GRCg6a) by HISAT2 (v2.1.0) (Kim et al., 2019) and Bowtie (v2.2.9) (Langmead and Salzberg, 2012). The mapped reads of each sample were assembled by Cufflinks (v2.2.1) (Trapnell et al., 2010).

The pipelines for lncRNAs identification were as follows: 1) transcripts that were merged or were similar to the known gallinaceous mRNAs and other small RNAs (rRNA, snRNA, snoRNAs, tRNAs, pre-miRNA, pseudogenes) were removed using Cuffcompare software (Ghosh and Chan, 2016). 2) the transcripts, annotated as “i (a transfrag falling entirely within a reference intron),” “u (unknown, intergenic transcript),” “x (exonic overlap with reference on the opposite strand)” or “o (generic exonic overlap with a reference transcript)” by the Cuffcompare software, were left for the next filter. 3) the remaining transcripts that contained a single exon and were shorter than 200 bp were removed. 4) The remaining transcripts were analyzed by the Coding Potential Calculator (CPC, v2.0) (Kang et al., 2017), Coding-Non-Coding-Index (CNCI, v2.0) (Sun et al., 2013), Pfam and Coding Potential Assessment Tool (CPAT, V2.0.0) (Wang et al., 2013). The transcripts that passed through all these stages were considered to be lncRNAs. Transcripts per million reads (TPM) was used to reflect the lncRNA expressional levels.

After the clean reads were aligned to the gallinaceous reference genome, the junctions of the unmapped reads were identified using a back-splice algorithm. Then, the Findcirc software was used to predict the circRNAs (Memczak et al., 2013). MMapped back splicing junction reads per million mapped reads (RPM) was used to reflect the expressional levels of circRNAs.

The DESeq2 (v1.10.1) software (Love et al., 2014) was used to detect differentially expressed mRNAs (DEMs), miRNAs (DEMIs), lncRNAs (DELs), and circRNAs (DECs) with *P*_adjust_ < 0.05 and |log_2_FoldChange| ≥ 1.

### Gene ontology and kyoto encyclopedia of genes and genomes enrichment analysis

Gene Ontology (GO) analyses were performed for DEMs and target genes using Goatools (v0.6.5) software. Kyoto Encyclopedia of Genes and Genomes (KEGG) pathway analyses for DEMs and target genes were carried out with the DAVID tool. The results with *P*_adjust_ < 0.05 were considered to be significantly enriched.

### Construction of the lncRNA/circRNA-miRNA-mRNA regulatory network

To determine the possible interactions between miRNA with lncRNA, circRNA, and mRNA, the miRNA-lncRNA, miRNA-circRNA, and miRNA-mRNA interactions were predicted with miRanda software (John et al., 2004). The miRanda software was used to analyze the relationships between DEMIs and DELs, DECs, DEMs which predicted DEMIs targets based on sequence complementarity, conserved target sites, and free energy of formation. Only alignments with no mismatch in the positions, 2-8 in the 5′end and energies lower than −20 kcal/mol were retained. The potential lncRNA/circRNA-miRNA-mRNA network was constructed and visualized using Cytoscape software (v3.6.0) based on the ceRNA theory (Salmena et al., 2011).

### Validation of RNA-seq data by RT-qPCR

Three DELs, DECs, DEMs, and DEMIs from the ceRNA network were selected to validate the results of RNA-seq by RT-qPCR, respectively. First-strand cDNA was synthesized with random hexamers (for mRNA, lncRNA, and circRNA) and stem-loop RT primers (for miRNA) by the PrimeScript RT reagent Kit (TaKaRa, Dalian, China). The glyceraldehyde-3-phosphate dehydrogenase (GAPDH, for mRNA, lncRNA, and circRNA) and 5S rRNA (for miRNA) were used as endogenous controls for normalization. All primers used in RT-qPCR are shown in Supplementary Table S1. All reactions were carried out in the CFX96 qPCR system (Bio-Rad, United States) with triplicate reactions for each sample. The quantification of relative expression of lncRNAs, circRNAs, mRNAs, and miRNAs was performed using the 2^−ΔΔCt^ method.

## Results

### Comparison of histological characteristics of muscle fiber

**FIGURE 1:**
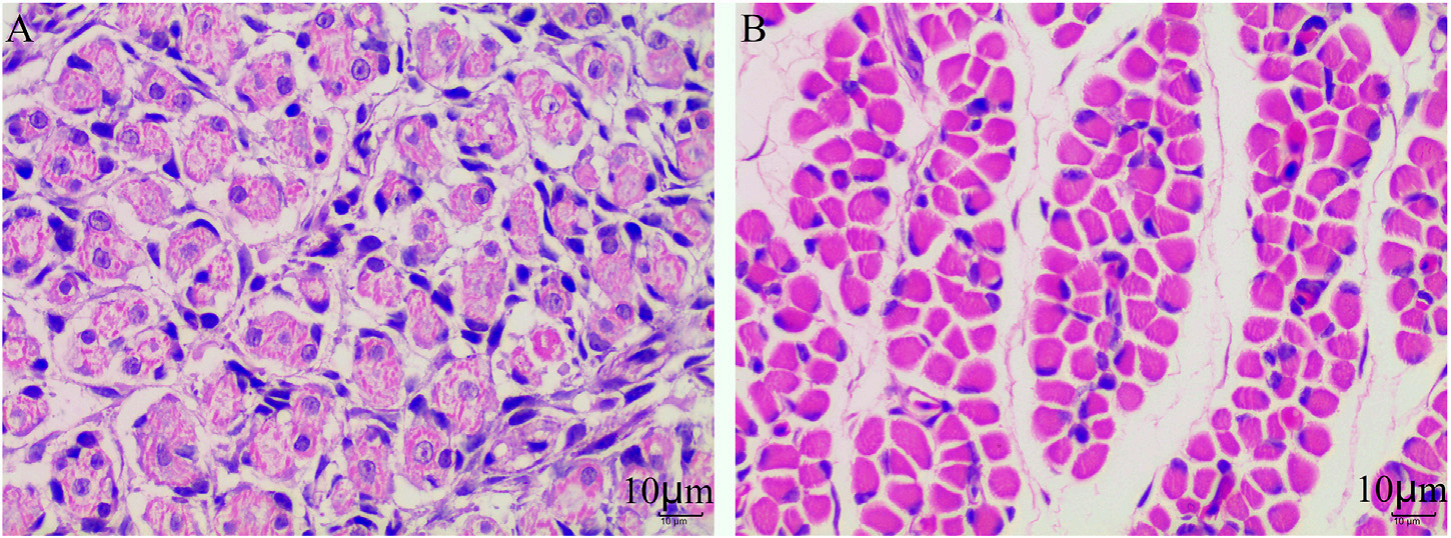
Muscle fiber characteristics of Tibetan chicken at E10 and E18. **(A)** H&E staining of the leg muscle cross-section from E10. **(B)** H&E staining of the leg muscle cross-section from E18.

To preferably understand the different performance of skeletal muscle fibers between E10 and E18, we used the muscle section following H&E staining to observe and analyze the morphology of the muscle fibers. The developmental muscle fibers are unclear and incomplete at the E10 stage which is in the mixed phase of myoblast proliferation and differentiation (Figure 1A). At the E18 stage, the muscle fibers have developed completely and the number of muscle fibers has been determined (Figure 1B).

### Overview of long-RNA sequencing

To obtain a global view of the Tibetan chicken leg muscle transcriptome and identify the lncRNA, circRNA, and mRNA transcripts related to the two developmental stages, six libraries were constructed and then sequenced. A total of 710.03 M (million) raw reads were obtained and an average of 118.34 M of raw reads per library. 704.07 M (99.16%) of raw reads were filtered and preserved as clean reads for the following analyses, where an average of 117.35 M clean reads per library (Supplementary Tables S2, S3).

**FIGURE 2:**
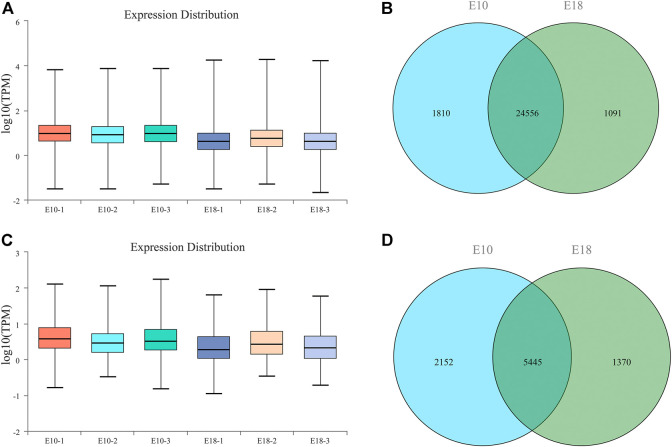
Overview of long RNA sequencing in the leg muscle of Tibetan chicken. **(A)** The boxplot of TPM (transcripts per million reads) for known protein-coding transcripts. **(B)** The Venn diagram of detected mRNAs identified in E10 and E18 group. **(C)** The boxplot of TPM (transcripts per million reads) for known lncRNA transcripts. **(D)** The Venn diagram of detected lncRNAs identified in E10 and E18 group.

In all the sequencing libraries, a total of 27,457 protein-coding transcripts including 25,768 known and 1,689 novel protein-coding transcripts were identified. The transcripts abundances were quantified by TPM, the average expression level of 25,768 known protein-coding transcripts was 15.90 (Figure 2A). We detected 26,366 and 25,647 protein-coding transcripts in the whole transcriptome of E10 and E18 group, respectively. A total of 24,556 protein-coding transcripts were co-expressed in E10 and E18, while 1,810 and 1,091 protein-coding transcripts were specifically expressed in E10 and E18, respectively (Figure 2B).

There were 8,967 lncRNA transcripts including 7,883 known and 1,084 novel protein-coding transcripts in total. 7,597 lncRNA transcripts were detected in the whole transcriptome of E10, and 6,815 lncRNA transcripts in E18. The average expression level of known lncRNA transcripts (1.93) was lower than known protein-coding transcripts (15.90) (Figure 2C). A total of 5,445 lncRNA transcripts were co-expressed in E10 and E18, while 2,152 and 1,370 lncRNA transcripts were specifically expressed in E10 andE18, respectively (Figure 2D).

### Analysis of DEMs

**FIGURE 3:**
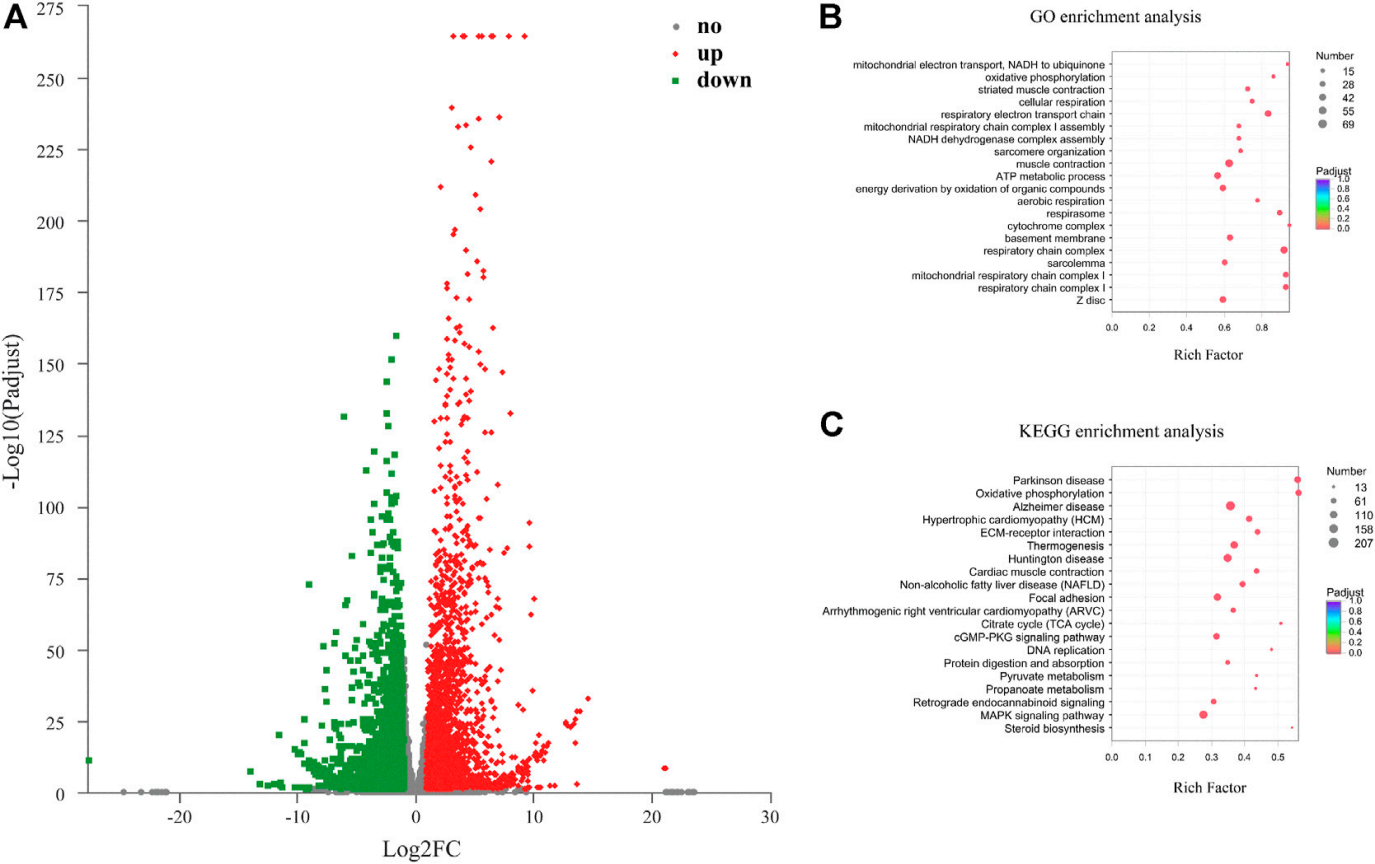
GO and KEGG pathway analysis of the differentially expressed mRNAs (DEMs) in the chicken leg muscle. **(A)** Volcano plot of 6,583 DEMs. **(B)** Top 20 significantly enriched GO terms for DEMs. **(C)** Top 20 significantly enriched pathways for DEMs.

Overall, 6,583 DEMs including 3,055 down-regulated (46.41%) and 3,528 up-regulated (53.59%) were discovered between the two developmental stages (Figure 3A). The top 20 up-regulated and down-regulated mRNAs were summarized in Table 1. GO and KEGG pathway enrichment analysis were performed to identify biological functions of DEMs. GO analysis of the DEMs showed that respiratory electron transport chain, muscle system process, striated muscle contraction, generation of precursor metabolites and energy, ATP metabolic process, regulation of cellular component movement, and cell cycle process were the most abundant terms in the biological process category. In the cellular component category, respirasome, mitochondrial respiratory chain complex I, and NADH dehydrogenase complex were the top three terms, while NADH dehydrogenase activity, actin binding, cytoskeletal protein binding, and NADH dehydrogenase (quinone) activity were the most abundant terms in the molecular function category (Figure 3B; Supplementary Table S4). Pathway analysis indicated that the DEMs were significantly enriched in 28 KEGG pathways, in which several pathways were related to skeletal muscle development, such as cell cycle, DNA replication, MAPK signaling pathway, cGMP-PKG signaling pathway, and Arginine biosynthesis (Figure 3C; Supplementary Table S5).

**TABLE 1:** The top 20 up-regulated and down-regulated mRNAs.

Category	Transcript id	Gene id	Gene symbol	*P*_adjust_
Up	ENSGALT00000099192	ENSGALG00000032619	SYPL2	1.58E-215
	ENSGALT00000013599	ENSGALG00000008351	CAV3	8.42E-214
	ENSGALT00000059202	ENSGALG00000006753	ATP5C1	9.88E-197
	ENSGALT00000043595	ENSGALG00000026553	—	1.15E-195
	ENSGALT00000006252	ENSGALG00000003925	MYPN	1.79E-193
	ENSGALT00000011524	ENSGALG00000007114	APOA1	1.67E-185
	ENSGALT00000046346	ENSGALG00000012211	PDLIM5	1.17E-175
	MSTRG.7615.2	ENSGALG00000037443	CRAT	3.76E-175
	ENSGALT00000049033	ENSGALG00000043287	CAV1	1.61E-163
	ENSGALT00000005164	ENSGALG00000003261	PTRF	3.52E-158
Down	ENSGALT00000039950	ENSGALG00000003475	ACLY	4.80E-106
	ENSGALT00000000816	ENSGALG00000000585	MYL4	1.34E-105
	ENSGALT00000020914	ENSGALG00000012821	TUBB2B	2.87E-103
	ENSGALT00000056361	ENSGALG00000003922	TOP2A	6.52E-100
	ENSGALT00000013437	ENSGALG00000008256	BLM	1.49E-99
	MSTRG.20698.2	ENSGALG00000008367	MDK	7.29E-99
	ENSGALT00000073565	ENSGALG00000008452	IFT122	1.35E-94
	ENSGALT00000036887	ENSGALG00000015358	MYH15	6.00E-94
	ENSGALT00000003987	ENSGALG00000002531	SF3B3	1.80E-93
	ENSGALT00000021124	ENSGALG00000012952	BASP1	1.09E-91

### Analysis of DELs and DECs

There are 695 lncRNA transcripts that were differentially expressed between the two developmental stages, including 187 that were down-regulated (26.91%) and 508 that were up-regulated (73.09%) in E18 (Supplementary Figure S1A). The top 20 up-regulated and down-regulated lncRNAs were summarized in Table 2. We predicted the potential cis and trans target DEMs of lncRNAs for investigating the function of DELs. For the cis action of DELs, protein-coding genes 10 and 100 kb upstream and downstream of the DELs were searched, respectively. The results showed 695 DELs corresponding to 133 DEMs within a range of 10 kb, as well as 272 DEMs within a range of 100 kb (Supplementary Table S6). On the other hand, the trans role of 695 DELs in protein-coding genes was examined based on their expression correlation coefficient (Pearson correlation ≥0.95 or ≤ −0.95). A total of 25,290 interaction relationships were detected in the trans form between 695 DELs and 6,583 DEMs in the chicken genome (Supplementary Table S7).

**TABLE 2:** The top 20 up-regulated and down-regulated lncRNAs.

Category	LncRNA id	Log_2_FC(E18/E10)	*P*_adjust_
Up	MSTRG.22805.1	4.360492	6.6E-131
	MSTRG.5174.3	4.006953	4.8E-101
	MSTRG.22803.1	4.511624	2.26E-71
	ENSGALT00000101377	7.021484	1.4E-56
	MSTRG.7257.18	2.278456	1.75E-43
	MSTRG.7257.17	2.040989	4.72E-30
	MSTRG.17517.1	2.607342	3.68E-27
	ENSGALT00000096941	2.909254	2.21E-26
	MSTRG.7257.16	3.693853	3.78E-26
	ENSGALT00000096019	2.975009	1.12E-24
Down	ENSGALT00000107394	−1.67209	6.65E-29
	MSTRG.18494.1	−1.25628	9.32E-21
	NONGGAT000245.2	−2.89597	5.18E-19
	ENSGALT00000102283	−1.90458	9.08E-18
	ENSGALT00000104731	−2.45094	6.52E-17
	ENSGALT00000093279	−1.67424	1.34E-16
	ENSGALT00000092976	−1.01148	1.39E-15
	ENSGALT00000106965	−1.87355	6.05E-14
	NONGGAT002481.2	−2.93494	6.06E-13
	ENSGALT00000102361	−4.04126	5.02E-12

Functional analysis showed that these target genes were significantly enriched in 504 GO terms (350 biological process terms, 91 molecular function terms, and 63 cellular component terms). Many GO terms were related to regulation of muscle system process, muscle fiber development, muscle cell development, muscle structure development, and muscle tissue development (Supplementary Figure S1B and Supplementary Table S8). In addition, the target genes were enriched in 23 pathways, some of which were associated with muscle development including DNA replication, ECM-receptor interaction, cell cycle, p53 signaling, glycine, serine, and threonine metabolism pathways (Supplementary Figure S1C and Supplementary Table S9). These results indicated that lncRNAs took part in several biological processes in the development of chicken muscle.

There were 1,906 DECs including 1,224 down-regulated (64.22%) and 682 up-regulated (35.78%) were discovered between the two developmental stages (Supplementary Figure S2A). The top 20 up-regulated and down-regulated circRNAs were summarized in Table 3. We performed GO and pathway enrichment analysis of host genes to identify biological functions of DECs. These host genes were mainly associated with GO terms including regulation of cell differentiation, developmental process, regulation of cell morphogenesis, and ncRNA-mediated regulation of translation (Supplementary Figure S2B and Supplementary Table S10). The KEGG results revealed that the enriched pathways involved Wnt signaling pathway and MAPK signaling pathway (Supplementary Figure S2C and Supplementary Table S11).

**TABLE 3:** The top 20 up-regulated and down-regulated circRNAs.

Category	CircRNA id	Host gene id	Log_2_FC(E18/E10)	*P*_adjust_
Up	18_616566_650758	—	6.604281	0
	18_568737_614281	—	6.167016	1.1E-282
	18_503467_567696	—	2.950716	1.7E-225
	2_14724132_14730427	ENSGALG00000007331	1.424465	2.6E-219
	14_8816217_8818041	ENSGALG00000030546	3.528837	3.5E-153
	2_14715253_14730427	ENSGALG00000007331	2.531163	2.2E-122
	7_15254259_15256437	—	5.657796	2.3E-119
	26_5070548_5072732	ENSGALG00000003498	1.893414	5.4E-119
	5_25346596_25348346	ENSGALG00000052873	1.428139	1.2E-108
	7_4707923_4710257	ENSGALG00000003862	1.418158	4.05E-82
Down	6_31878072_31892396	ENSGALG00000009495	−5.08502	7.4E-141
	3_88394208_88403946	—	−2.11108	5.54E-74
	18_385835_464595	—	−4.0474	1.54E-49
	15_6681798_6681904	ENSGALG00000004818	−9.10084	4.59E-45
	4_1306777_1310899	—	−2.0855	4.18E-42
	22_360264_375976	ENSGALG00000030603	−2.26215	3.92E-40
	1_195385200_195385756	—	−2.99086	1.45E-31
	1_62147456_62157399	—	−2.67989	3.44E-31
	10_17683216_17696640	ENSGALG00000038688	−3.54978	5.35E-26
	10_17668028_17696640	ENSGALG00000038688	−2.11303	7.33E-26

### Overview of Small-RNA sequencing

**FIGURE 4:**
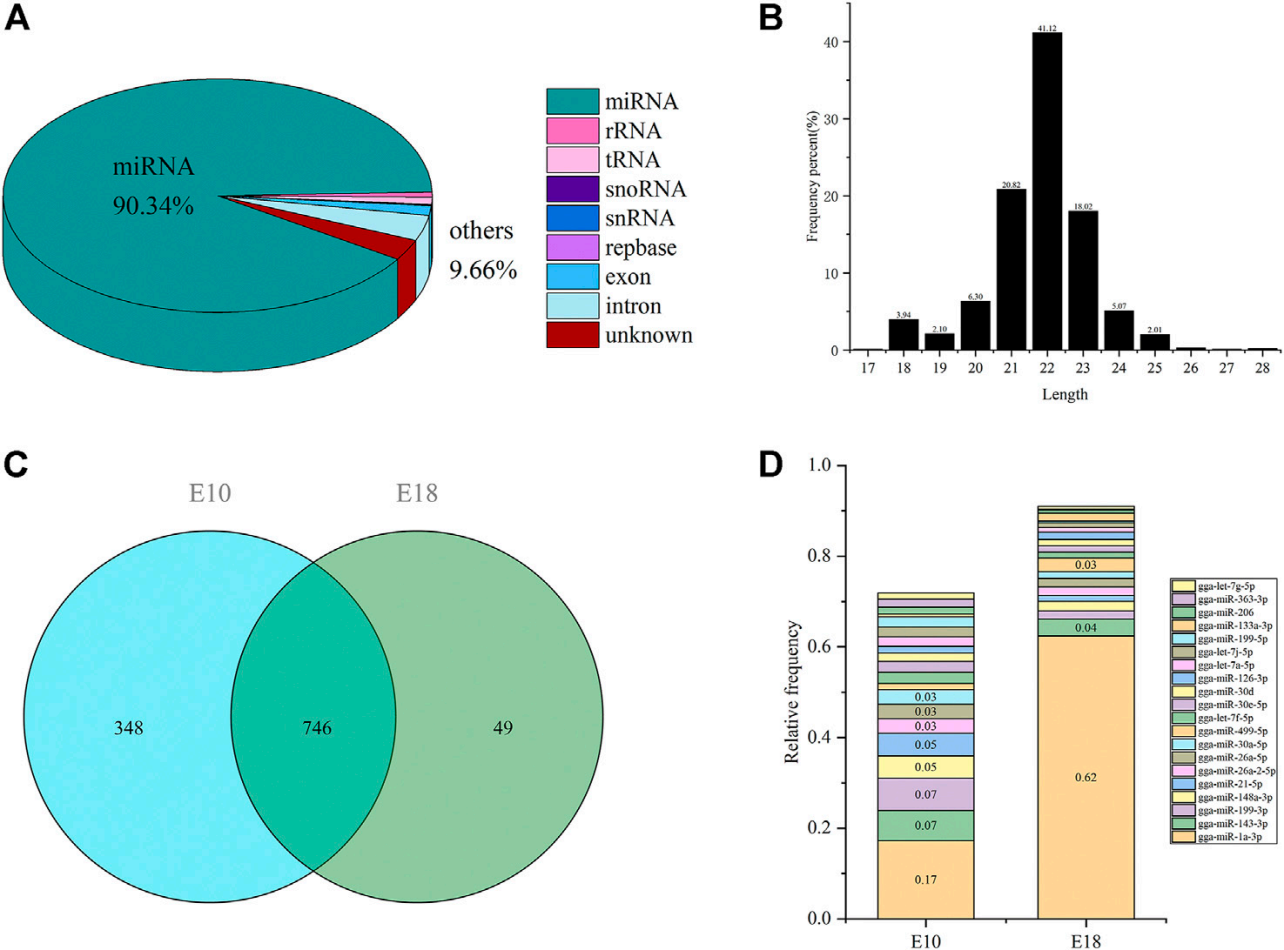
Overview of small RNA sequencing in the leg muscle of Tibetan chicken. **(A)** Portions of small RNA types in the useful reads. The percent of miRNA is 90.34%, and the other 9.66% included rRNA, tRNA, snRNA, snoRNA, repbase, exon, and intron. **(B)** Length distribution of all miRNAs. **(C)** The Venn diagram of detected miRNAs identified in E10 and E18 group. **(D)** The relative proportion of the top 20 miRNAs in the total amount of miRNAs.

As shown in Supplementary Tables S11, S12, six libraries were constructed and sequenced, resulting in a total of 67.21 M raw reads, and an average of 11.20 M of raw reads was obtained per library. 63.46 M (94.43%) of raw reads were filtered and preserved as useful reads for the subsequent analyses, where an average of 10.58 M useful reads per library (Supplementary Tables S12, S13). After alignment with small RNAs in the reference genome, miRBase and Rfam, we identified 90.34% miRNAs, and other small RNAs (9.66%) including rRNA, tRNA, snRNA, snoRNA as well as repbase (Figure 4A). We identified a total of 1,143 miRNAs in six small RNA libraries, and the length of most miRNAs were 18–24 nucleotides (Figure 4B). Total 1,094 and 795 miRNAs were detected in E10 and E18, respectively. A total of 746 miRNAs were co-expressed in E10 and E18, while 348 and 49 miRNAs were specifically expressed in E10 and E18, respectively (Figure 4C). Twenty mature miRNAs with the highest expression comprised 71.88 and 91.02% of all miRNAs in E10 and E18 (Figure 4D), of which gga-miR-1a-3p, gga-miR-143-3p, gga-miR-199-3p, gga-miR-148a-3p, and gga-miR-21-5p accounting for 56% of the total distribution, were the most abundant miRNAs.

### Analysis of DEMIs

There were 130 DEMIs including 59 that were up-regulated (45.38%) and 71 that were down-regulated (54.61%) between the two developmental stages (Supplementary Figure S3A). The top 20 up-regulated and down-regulated miRNAs were summarized in Table 4. A total of 3,834 target DEMs (Supplementary Table S14), 440 target DELs (Supplementary Table S15), and 1,581 target DECs (Supplementary Table S16) for DEMIs were identified. GO and pathway enrichment analysis were performed on target DEMs to identify biological functions of DEMIs. These genes were mainly associated with muscle system process, myofilament, striated muscle cell development, structural constituent of muscle, skeletal muscle thin filament assembly, muscle cell development, skeletal muscle myosin thick filament assembly, and muscle fiber development (Supplementary Figure S3B and Supplementary Table S17). The result of KEGG pathway analysis indicated the enriched pathways involved DNA replication, ECM-receptor interaction, valine, leucine and isoleucine degradation, and arginine biosynthesis (Supplementary Figure S3C and Supplementary Table S18).

**TABLE 4:** The top 20 up-regulated and down-regulated miRNAs.

Category	MiRNA id	Log_2_FC(E18/E10)	*P*_adjust_
Up	gga-miR-133a-5p	2.732811	2.3E-103
	gga-miR-133a-3p	3.584224	2.86E-98
	gga-miR-133c-3p	2.607947	1.53E-65
	gga-miR-191-5p	2.271249	1.76E-63
	gga-let-7b	2.793175	4E-58
	gga-miR-1a-3p	3.904566	4E-58
	gga-miR-145-5p	1.736813	1.74E-57
	gga-miR-30b-5p	2.121494	4.18E-43
	gga-miR-23b-3p	1.898131	1.2E-36
	gga-miR-26a-2-5p	1.244917	5.64E-26
Down	gga-miR-301a-5p	−2.32789	2.54E-29
	gga-miR-218-5p	−1.55071	4.63E-18
	gga-miR-92-5p	−1.7563	2.83E-17
	gga-miR-181b-1-3p	−1.74845	9.05E-17
	gga-miR-205b	−4.71672	4.24E-16
	gga-miR-222a	−1.61754	4.87E-16
	gga-miR-1677-3p	−2.4034	5.05E-16
	gga-miR-1662	−2.32382	5.81E-15
	gga-miR-1677-5p	−2.60366	1.31E-12
	gga-miR-18b-5p	−1.47996	1.91E-12

### Construction of miRNA-gene-pathway relationship network

**FIGURE 5:**
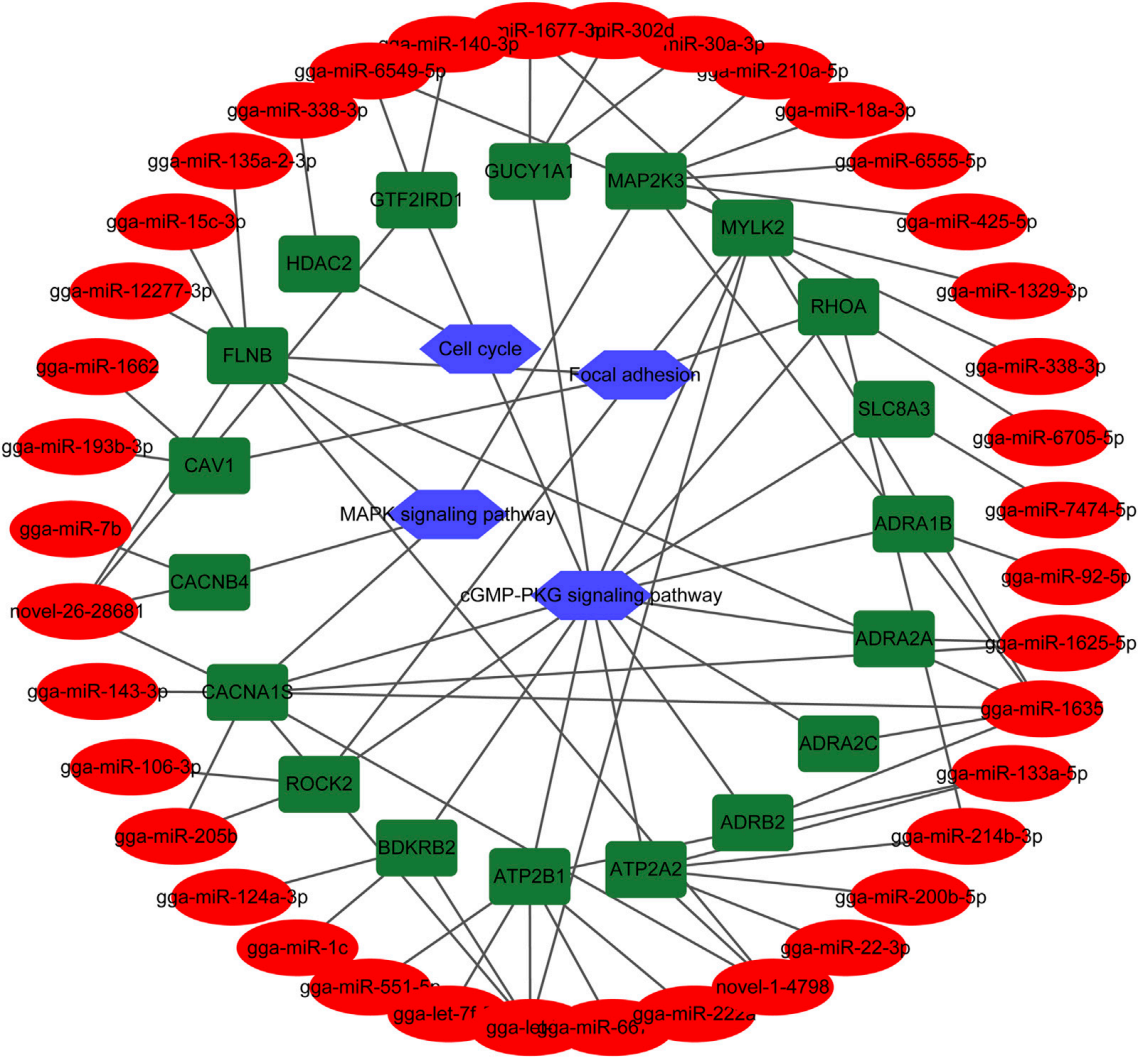
The miRNA-gene-pathway network between 19 DEMs involved in muscle system process, and their corresponding pathways and DEMIs. Blue hexagon, green round rectangle and red ellipse indicate pathway, gene and miRNA, respectively.

Among the DEMs, 19 of them involved in muscle system process, ECM-receptor interaction, Focal adhesion, cGMP-PKG signaling pathway, MAPK signaling pathway, and cell cycle corresponded with 39 DEMIs (Table 5). Rho associated coiled-coil containing protein kinase 2 (*ROCK2*), calcium voltage-gated channel subunit alpha1 S (*CACNA1S*), filamin B (*FLNB*), myosin light chain kinase 2 (*MYLK2*), and ras homolog family member A (*RHOA*) genes participate in multiple pathways. To further understand and visualize the interactions and investigate the function of corresponding DEMIs, a miRNA-gene-pathway network was constructed (Figure 5) using the data from Table 5. The potential functions of a few miRNAs were identified by the interaction analysis in the skeletal muscle, for example, gga-miR-338-3p may play important roles in cell proliferation, gga-let-7b, gga-miR-1625-5p, gga-miR-1635, gga-miR-1677-3p, gga-miR-205b, gga-miR-214b-3p, gga-miR-338-3p, gga-miR-6549-5p, novel-1-4,798, and novel-26-28681were closely associated with multiple pathways including cGMP-PKG signaling pathway, focal adhesion and MAPK signaling pathway.

**Table 5:** 19 DEMs involved in muscle system process and their corresponding pathways and related DEMIs.

DEMs	Pathway	Related DEMIs
*ADRA1B*	cGMP-PKG signaling pathway	gga-miR-92-5p
*ADRA2A*	cGMP-PKG signaling pathway	gga-miR-1625-5p
*ADRA2C*	cGMP-PKG signaling pathway	gga-miR-1635
*ADRB2*	cGMP-PKG signaling pathway	gga-miR-1635
*ATP2A2*	cGMP-PKG signaling pathway	gga-miR-133a-5p, gga-miR-214b-3p, gga-miR-200b-5p, gga-miR-22-3p, novel-1-4798
*ATP2B1*	cGMP-PKG signaling pathway	gga-miR-222a, gga-miR-6670-5p, gga-let-7b, gga-let-7f-5p, gga-miR-551-5p, gga-miR-133a-5p
*BDKRB2*	cGMP-PKG signaling pathway	gga-miR-1c, gga-let-7b, gga-miR-124a-3p
*ROCK2*	cGMP-PKG signaling pathway	
	Focal adhesion	gga-miR-205b, gga-miR-106-3p
*CACNA1S*	cGMP-PKG signaling pathway	
	MAPK signaling pathway	gga-miR-1625-5p, gga-let-7b, gga-miR-205b, gga-miR-1635, gga-miR-143-3p, novel-1-4798, novel-26-28681
*CACNB4*	MAPK signaling pathway	gga-miR-7b, novel-26-28681
*CAV1*	Focal adhesion	gga-miR-193b-3p, gga-miR-1662
*FLNB*	Focal adhesion	
	MAPK signaling pathway	gga-miR-1635, gga-miR-12277-3p, gga-miR-15c-3p, gga-miR-135a-2-3p, novel-1-4798, novel-26-28681
*HDAC2*	Cell cycle	gga-miR-338-3p
*GTF2IRD1*	cGMP-PKG signaling pathway	gga-miR-6549-5p, gga-miR-140-3p, novel-26-28681
*GUCY1A1*	cGMP-PKG signaling pathway	gga-miR-1677-3p, gga-miR-302d, gga-miR-30a-3p
*MAP2K3*	MAPK signaling pathway	gga-miR-210a-5p, gga-miR-18a-3p, gga-miR-338-3p, gga-miR-1635, gga-miR-210a-5p, gga-miR-6555-5p, gga-miR-425-5p
*MYLK2*	cGMP-PKG signaling pathway	
	Focal adhesion	gga-miR-1329-3p, gga-let-7b, gga-miR-6549-5p, gga-miR-1635
*RHOA*	cGMP-PKG signaling pathway	
	Focal adhesion	gga-miR-214b-3p, gga-miR-1677-3p, gga-miR-6705-5p
*SLC8A3*	cGMP-PKG signaling pathway	gga-miR-7474-5p

### Construction of ceRNA regulatory network

**FIGURE 6:**
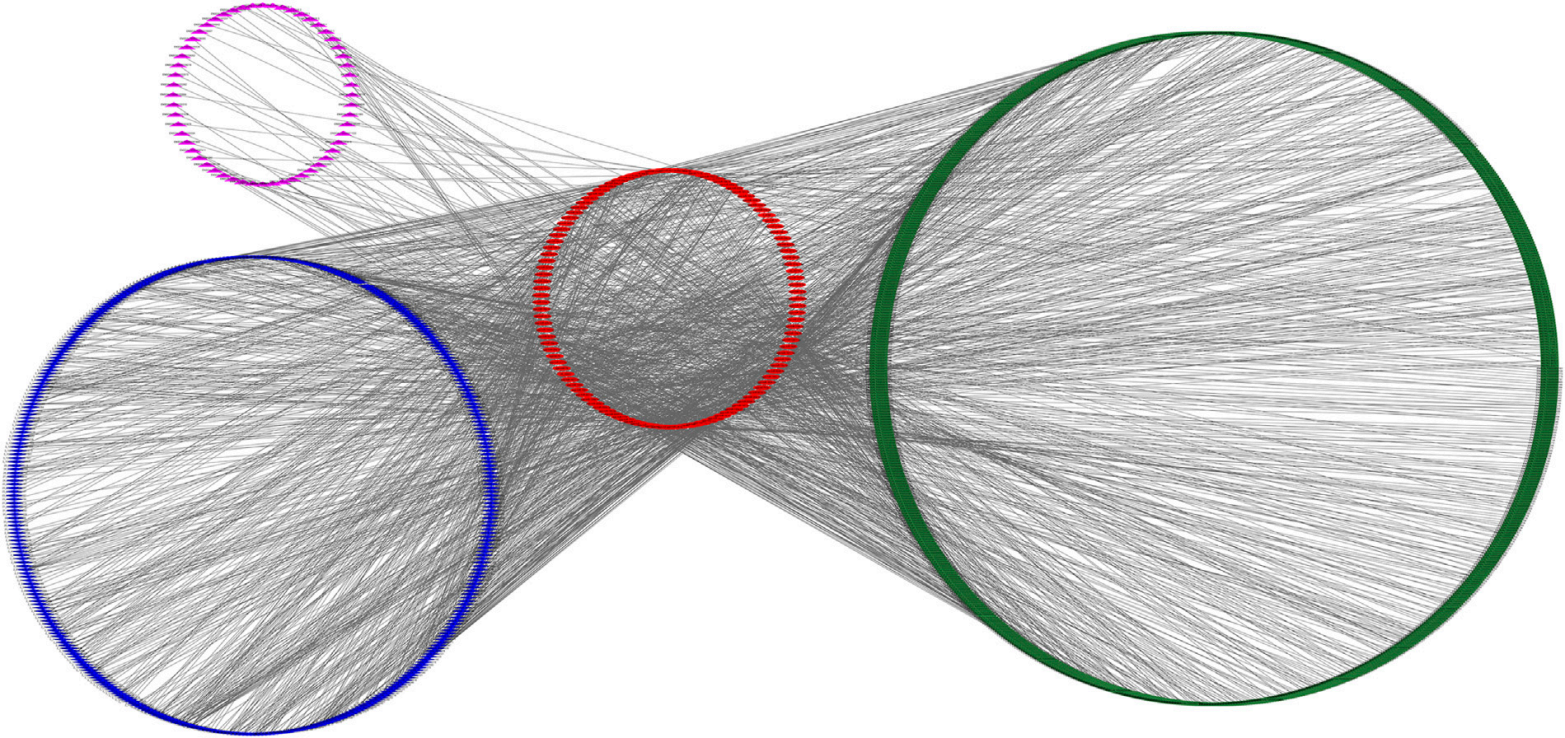
An overview of the competing endogenous RNA (ceRNA) network. Pink triangle, blue rhombus, red ellipse, and green round rectangle indicate circRNA transcript, lncRNA transcript, miRNA and mRNA transcript, respectively.

Based on the data of 6,583 DEMs, 695 DELs, 1,906 DECs, and 130 DEMIs, we identified putative miRNA-mRNA, miRNA-lncRNA, and miRNA-circRNA interactions. In total, we obtained 11,326 miRNA-mRNA, 1,266 miRNA-lncRNA, and 11,905 miRNA-circRNA interaction pairs. We next extracted miRNAs that paired with both lncRNA/circRNAs and mRNAs to construct the ceRNA network which included 115 miRNAs, 788 mRNAs, 56 lncRNAs, 398 circRNAs, and 2,161 interaction pairs (Figure 6).

**FIGURE 7:**
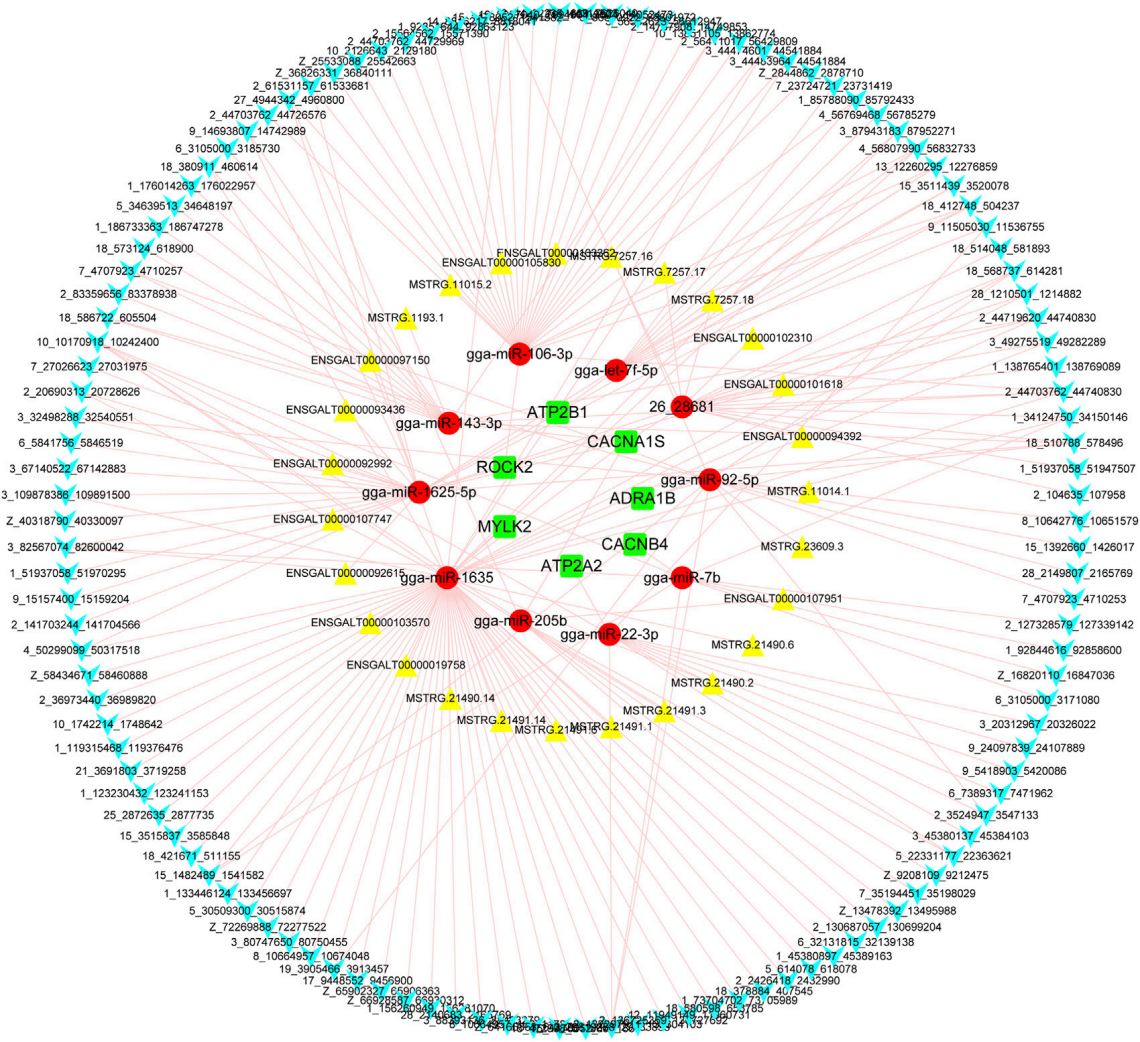
The predicted interaction between circRNA transcript, lncRNA transcripts and *ADRA1B*, *ATP2A2*, *ATP2B1*, *CACNA1S*, *CACNB4*, *MYLK2*, and *ROCK2* genes. Blue polygon, yellow triangle, red ellipse, and green round rectangle indicate circRNA transcript, lncRNA transcript, miRNA, and mRNA, respectively.

The interactions between lncRNA/circRNAs and mRNAs were predicted through combining analysis of the ceRNA and miRNA-gene-pathway networks (Figure 7). These interactions referred to 123 circRNAs, 27 lncRNAs, 11miRNAs (gga-let-7f-5b, gga-miR-106-3p, gga-miR-143-3p, gga-miR-1625-5p, gga-miR-1635, gga-miR-193b-3p, gga-miR-205b, gga-miR-22-3p, gga-miR-7b, gga-miR-92-5p, and novel-26-28681), and 8 mRNAs (*ADRA1B*, *ATP2A2*, *ATP2B1*, *CACNA1S*, *CACNB4*, *MYLK2*, and *ROCK2*).

### Validation of RNA-seq data

**FIGURE 8:**
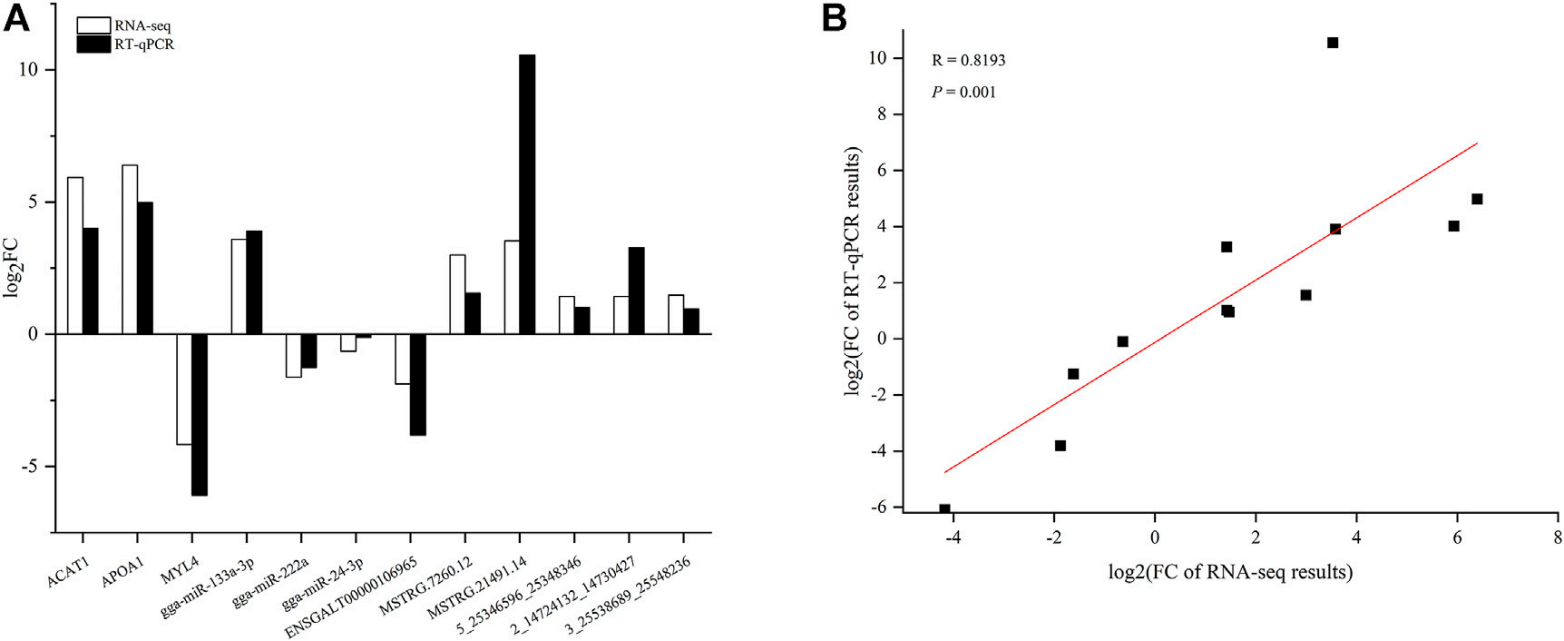
Validation of RNA-seq data using real time quantitative PCR (RT-qPCR) **(A)** and correlation between RNA-seq and RT-qPCR results **(B)**.

Three DEMs, DELs, DECs, and DEMIs from the ceRNA network were selected to validate the RNA-seq results using RT-qPCR, respectively. The results were consistent with the RNA-seq data (Figure 8A) and a high correlation was detected, with a Pearson’s correlation coefficient of 0.8193 (*p* < 0.01) (Figure 8B).

## Discussion

Chicken is an important agricultural animal that has become the largest consumer meat worldwide over the past 50 years (Petracci and Cavani, 2012). The production and quality of poultry meat are closely related to the development of skeletal muscle (Dransfield and Sosnicki, 1999). Leg muscle is a primary contributor to skeletal muscle and is directly related with the quantity and quality of meat. The exploration of molecular mechanisms underlying skeletal muscle development has been a focus in the field of poultry genetic breeding. Muscle growth is characterized by an increase in myoblast cell number through hyperplasia during the embryonic period of development, and the process is nearly complete at the time of hatch (Velleman, 2007). To obtain a full-scale picture of the transcriptome level changes that occur within the increase of myoblast cell number, whole transcriptome analysis was used to detect candidate gene function and their regulatory effectors. In total, 6,583 DEMs, 695 DELs, 1,906 DECs, and 130 DEMIs were detected between the two developmental stages of Tibetan chicken.

It is clear that RNA has a diverse set of functions and is more than just a messenger between gene and protein. Considering the differential expression of RNAs from these two stages, these RNAs might be associated with myogenesis, particularly thousands of ncRNAs are well-expressed with exquisite cell-type and tissue specificity (Mercer et al., 2008; Guttman et al., 2010). In the present study, we detected DEMs relevant to the development of skeletal muscle in the leg muscle of chicken at E18. For example, myogenin (MYOG), as an MRF, which has a unique role in fetal myogenesis, was decreased in chickens at E18. MYOG is indispensable for myogenic differentiation, and fetal myogenesis essentially fails in *MYOG*-null mice, with few differentiated myofibers present (Hasty et al., 1993; Nabeshima et al., 1993). Primary myogenesis is complete by E14.5, with all muscle groups largely established, and secondary myogenesis begins at approximately E16.5, with fetal myoblasts proliferating (Zammit, 2017). The differential expression of *MYOG* indicates that E10 and E18 were two different phases of developmental myogenesis. Notably, several detected DEMs were well-known related to muscle development. For example, caveolin 3 (*CAV3*) impairs skeletal muscle mitochondrial form and function (Shah et al., 2020). Myopalladin (*MYPN*) promotes muscle growth through modulation of the serum response factor pathway (Filomena et al., 2020). The myocyte enhancer binding factor 2 (MEF2) family including *MEF2A*, *MEF2B*, and *MEF2C* act synergistically with MyoD and myogenin to promote muscle differentiation and activation of muscle genes (Taylor and Hughes, 2017).

Muscle development is a complex and multi-stage process with many genes cooperatively involved in the regulation of each stage. It is also very conservative among vertebrates. The process can be divided into four major stages: somite differentiation into dermomyotome-containing myogenic precursors, myogenic precursor proliferation and differentiation into myoblasts, myoblast proliferation, determination, differentiation and fusion into myotube, and finally maturation (or differentiation) of myotubes into myofibers (Luo et al., 2013). Compared to the unclear and incomplete muscle fibers at E10, the complete and determined muscle fibers at E18 were closely related to the DEMs significantly enriched in many signal transduction pathways including focal adhesion, cGMP-PKG signaling, MAPK signaling, and cell cycle pathways, resulting in skeletal muscle development. In our study, we found increased expression of genes related to the above-mentioned pathways at E18, indicating that myoblast proliferation, differentiation, fusion into myotube, and finally maturation of myotubes into myofibers were mediated by the above pathways. Some of the pathways related to muscle development involved in DEMs such as MAPK signaling pathway were also found in the embryonic muscle development of the Chengkou Mountain Chicken (Ren et al., 2021). In addition, the target genes of miRNAs and lncRNAs were also found to be enriched in the mentioned pathways.

Expression of ATPase sarcoplasmic/endoplasmic reticulum Ca^2+^ transporting 2 (*ATP2A2*) and *CACNA1S* was increased. Intracellular Ca^2+^ is a critical coordinator of various aspects of cellular physiology. The Ca^2+^ adenosine triphosphatase SERCA encoded by *ATP2A2* controls of cell death and survival in various cell types (Nelson et al., 2016; Chemaly et al., 2018). The α1s subunit of the dihydropyridine receptor (DHPR α1s) encoded by *CACNA1S* controls skeletal muscle mass and morphogenesis (Piétri-Rouxel et al., 2010). We also found that the level of *MYLK2* and *ROCK2* were increased. The *MYLK2* gene which encodes skeletal muscle myosin light chain kinase regulates skeletal myogenesis by phosphorylation of MEF2C (Al Madhoun et al., 2011; Stull et al., 2011). Goetsch et al. (2014) found that *ROCK2* regulates directional myoblast migration through focal adhesion formation and maturation. Altogether, these results suggest that E10 to E18 are critical stages for myoblast proliferation, differentiation and finally maturation (or differentiation) of myotubes into myofibers.

In recent years, lncRNA and circRNA ceRNAs have received increased attention for their involvement in the development of skeletal muscle. For example, lncRNA 2310043L19Rik inhibits differentiation and promotes proliferation of myoblast by sponging miR-125a-5p (Li et al., 2020). As a ceRNA for miR-351-5p and miR-125b, lnc-mg mediates skeletal myogenesis by indirectly targeting lactamase-β (Zhu et al., 2017; Du et al., 2019). It was reported that circ-FoxO3 is a sponge of miR-138-5p which inhibits myoblast cells differentiation (Li et al., 2019a). CircMYBPC1 was identified to promote myoblast differentiation by directly binding miR-23a to relieve its inhibition on *MyHC* (Chen et al., 2021). Integrated analysis of lncRNA and mRNA expression profiles revealed that lncRNAs and target genes that might contribute to the regulation of different embryonic stages of skeletal muscle development in chicken (Li et al., 2016; Wu et al., 2022). Many miRNAs related to embryonic muscle development identified in Chengkou mountain chicken were also found in Tibetan chicken (Shi et al., 2022). We have not found research on circRNA sequencing related to embryonic muscle development in chicken. Our study showed expression profiles of lncRNA and circRNA transcripts in the chicken skeletal muscle at E10 and E18 by whole transcriptome sequencing. Through interaction analysis of DEMs, DELs, DECs, and DEMIs, we also discovered that lncRNA and circRNA could compete for miRNAs binding sites with mRNA and subsequently influence their expression. The genes associated with skeletal muscle development such as *ATP2A2*, *CACNA1S*, *MYLK2*, and *ROCK2* could be modulated by lncRNAs and circRNAs. These findings indicate that these lncRNAs and circRNAs could modulate multiple subsystems involved in the development skeletal muscle as regulators.

Integration of multi-omics can generate new knowledge that is not accessible by analysis of single datasets alone (Stanberry et al., 2013). Recent reports have described an intricate interplay among diverse RNA species, including protein-coding messenger RNAs and non-coding RNAs such as lncRNAs, pseudogenes and circRNAs (Tay et al., 2014). These RNA transcripts containing miRNA response elements (MREs) act as ceRNAs, communicate with and co-regulate each other by competing for binding to shared miRNAs. Our study constructed a ceRNA network by integrating multiple omics analyses, and subsequently detected lncRNA, circRNA, and miRNA highly relevant to skeletal muscle development.

In summary, we characterized and compared the expressional features of mRNAs, lncRNAs, circRNAs, and miRNAs in the skeletal muscle of the Tibetan chicken at E10 and E18 by RNA-seq. The DEMs, DELs, DECs, and DEMIs between the two stages involved in skeletal muscle development were identified and characterized. We conducted lncRNAs/circRNAs-miRNA-mRNA regulatory networks of the molecular mechanism for skeletal muscle development. The present study provided a new insight of the molecular mechanism underlying skeletal muscle development in chicken.

## Data Availability

The data presented in the study are deposited in the SRA repository, accession number "PRJNA758717".
