# Supplementary material for: Characterization of lncRNA/circRNA-miRNA-mRNA network to reveal potential functional ceRNAs in the skeletal muscle of chicken
Source: Front Physiol. 2022 Sep 29;13:969854. doi: 10.3389/fphys.2022.969854 (PMC9558166; doi:10.3389/fphys.2022.969854)
Supplement: Supplementary file 2 [file DataSheet1.docx]

Figure S1 GO and KEGG pathway analysis for the target DEMs of the differentially expressed lncRNAs (DELs) in the chicken leg muscle. (A) Volcano plot of 695 DELs. (B) Top 20 significantly enriched GO terms for the target DEMs of DELs. (C) Top 20 significantly enriched pathways for the target DEMs of DELs.





Figure S2 GO and KEGG pathway analysis for the host genes of the differentially expressed circRNAs (DECs) in the chicken leg muscle. (A) Volcano plot of 1,906 DECs. (B) Top 20 significantly enriched GO terms for the host genes of DECs. (C) Top 20 significantly enriched pathways for the host genes of DECs.





Figure S3 GO and KEGG pathway analysis for the target DEMs of the differentially expressed miRNAs (DEMIs) in the chicken leg muscle. (A) Volcano plot of 130 DEMIs. (B) Top 20 significantly enriched GO terms for the target DEMs of DEMIs. (C) Top 20 significantly enriched pathways for the target DEMs of DEMIs.
